# Supplementary material for: Effectors of anterior morphogenesis in C. elegans embryos
Source: Biol Open. 2023 Jul 5;12(7):bio059982. doi: 10.1242/bio.059982 (PMC10339035; doi:10.1242/bio.059982)
Supplement: Supplementary information [file biolopen-12-059982-s1.pdf]

Fig S1

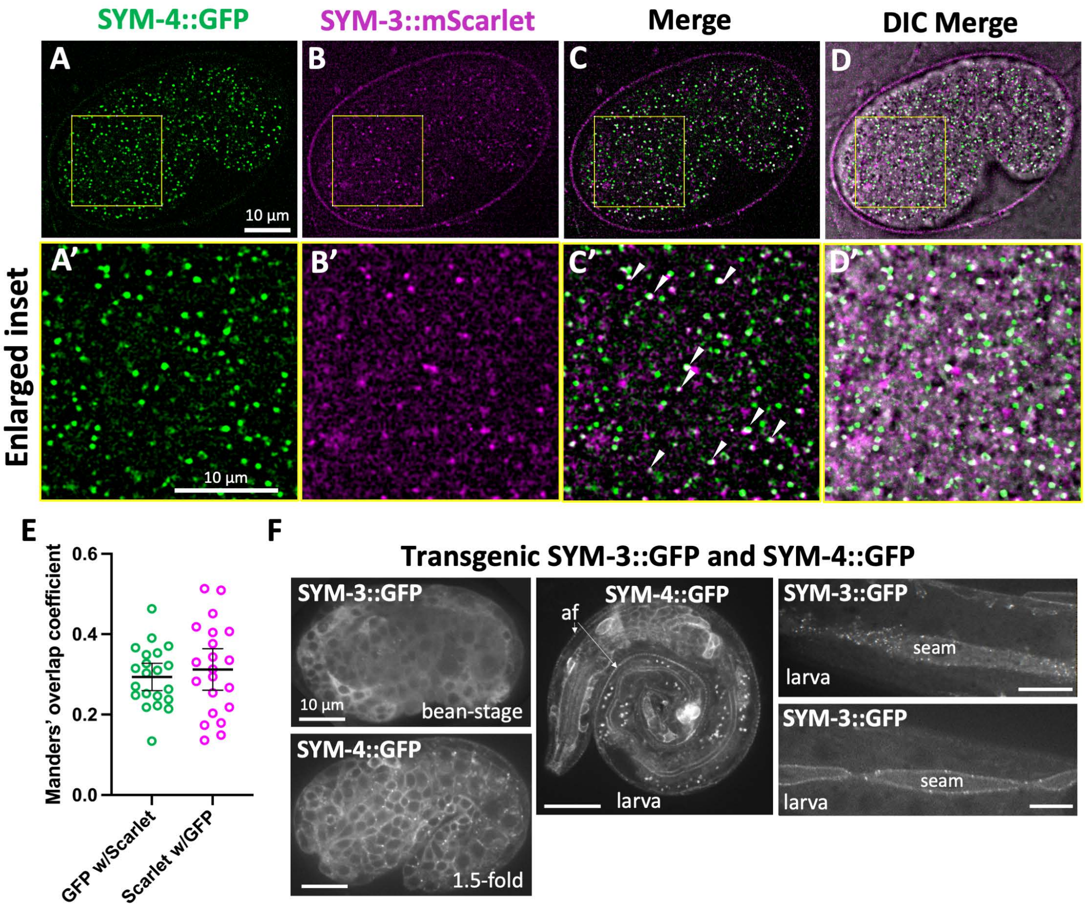

Fig. S1. Supplementary images of SYM-3 and SYM-4 expression

(A–D) Expression and colocalization of endogenously tagged SYM-3::mScarlet and SYM-4::GFP in early comma-stage embryos. Anterior is to the left, ventral is down. Arrowheads indicate puncta that overlap (white dots). (A'–D') Enlarged regions from (A–D) as indicated by the yellow boxes. (E) Manders' overlap coefficient for SYM-3::mScarlet and SYM-4::GFP embryos. (F) Images of bean-stage and 1.5-fold embryos and larvae expressing SYM-3::GFP and SYM-4::GFP recombineered fosmids from extrachromosomal arrays. Note broad expression and cytoplasmic localization of SYM-3 and SYM-4 including some puncta in SYM-4::GFP embryos and SYM-3::GFP seam cells. Autofluorescence (af) of the cuticle is indicated in the middle panel.

Fig S2

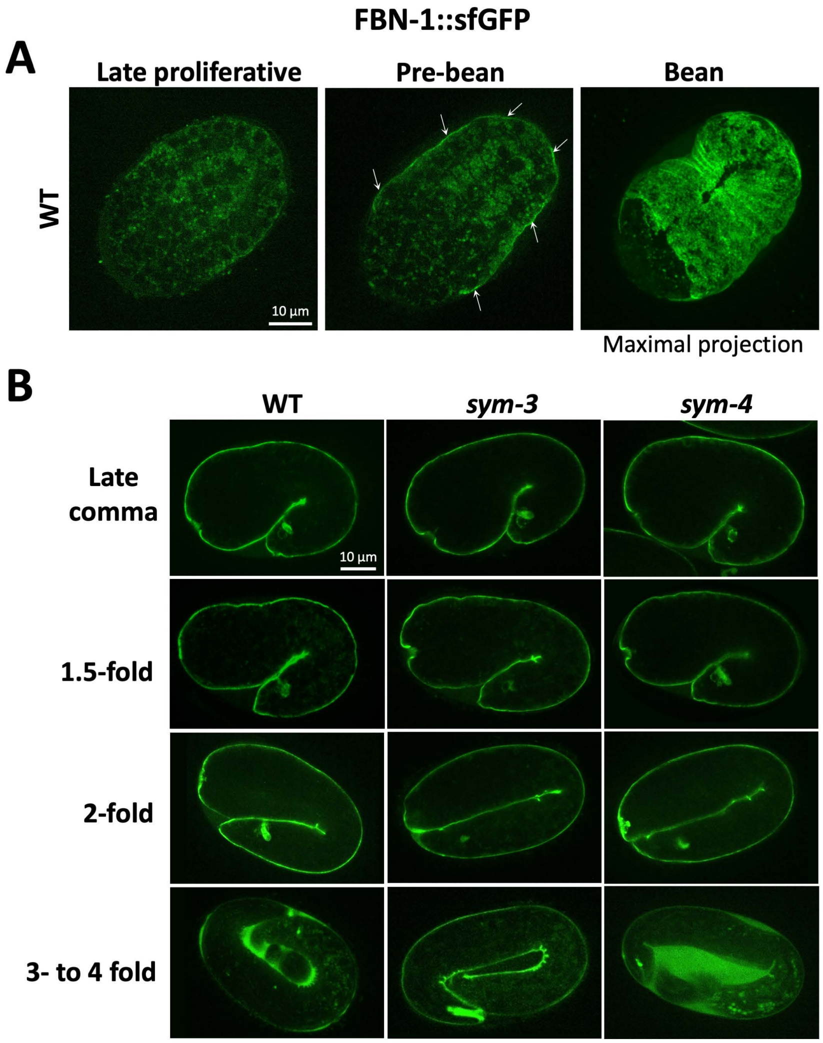

Fig. S2. Embryonic expression of FBN-1::sfGFP

(A) Endogenously tagged FBN-1::sfGFP in representative wild-type (WT) embryos at the indicated stages. Anterior is to the left, ventral is down. Arrows (middle panel) indicate the appearance of apical GFP, coincident with the onset of morphogenesis. Note FBN-1::sfGFP beginning to cover the apical surface in the maximal-projection image of the bean-stage embryo (rightmost panel) The absence of FBN-1::sfGFP signal in the anterior is consistent with a lack of anterior epidermal cells at this stage; epidermal cells complete migration to and containment of the anterior by the comma stage (Grimbert et al., 2021). (B) Representative FBN-1::sfGFP expression in wild-type, *sym-3*, and *sym-4* embryos at the indicated stages.

Biology Open • Supplementary information

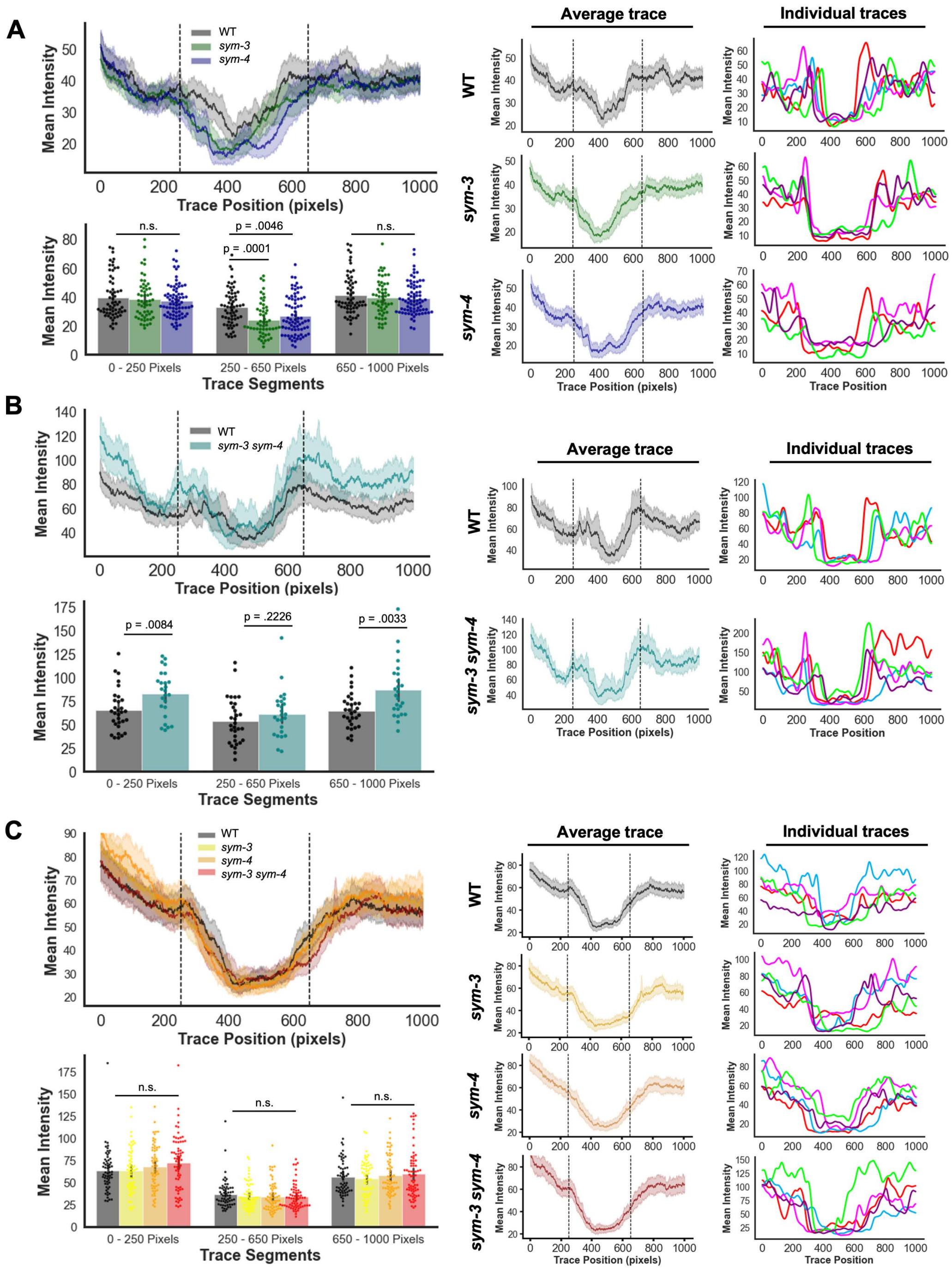

Fig. S3. Analysis of FBN-1::sfGFP and NOAH-1::mCherry anterior traces

Mean fluorescence intensities of (A, B) FBN-1::sfGFP and (C) NOAH-1::mCherry across 10-pixel wide by 1000-pixel long traces were carried out along the anterior apical regions of wild-type (WT), *sym-3*, *sym-4* and *sym-3 sym-4* embryos. Shaded areas represent the 95% confidence intervals for the averaged curves. Representative traces from 4–5 individual embryos of each genotype are also shown, which were smoothed using a gaussian filter. Quantification of trace intensities were carried out by segments as indicated (from dorsal to ventral; 0–250 pixels 250–650 pixels and 650–1000 pixels. P-values were calculated using a two-tailed Mann-Whitney test.

Biology Open • Supplementary information

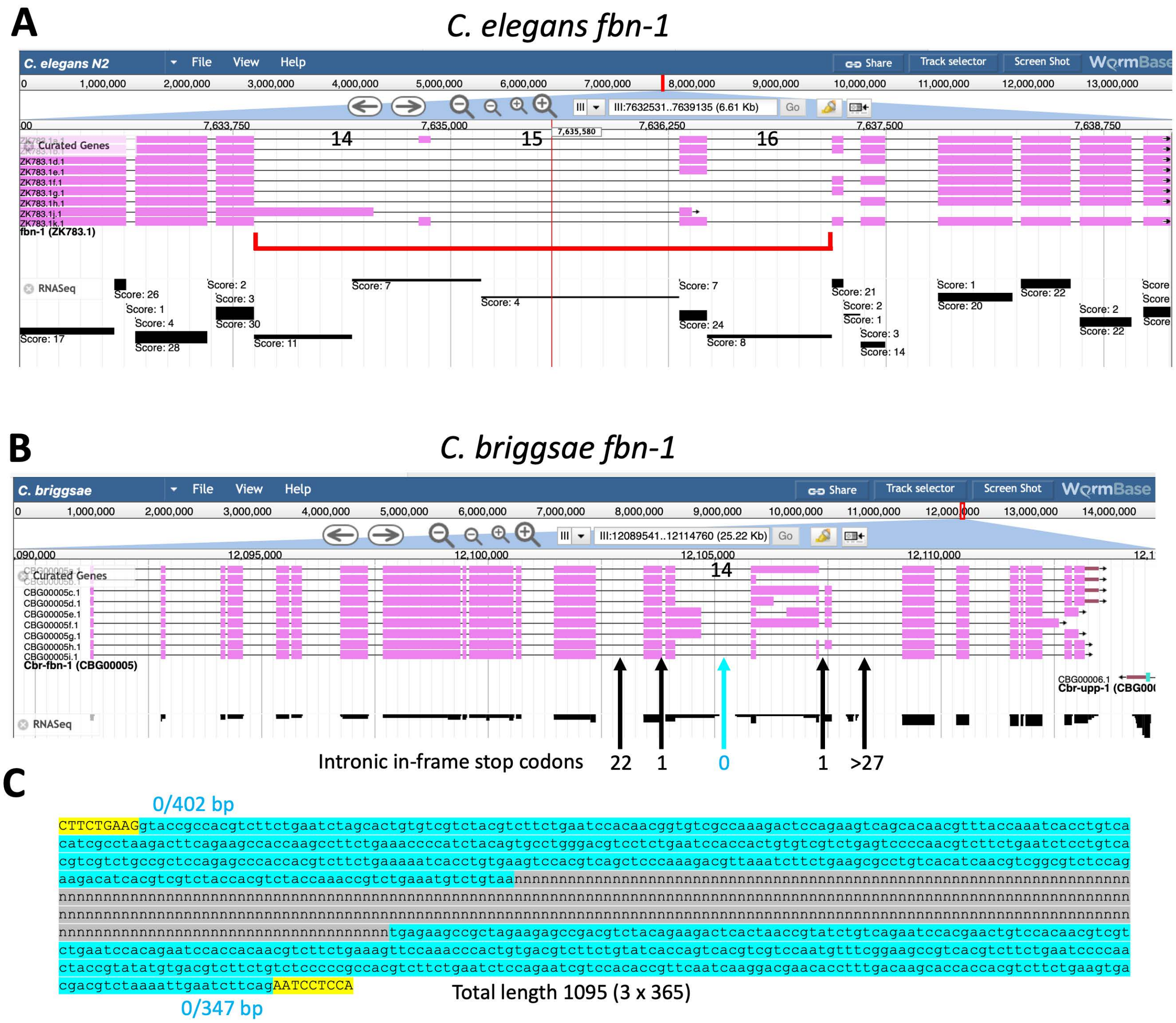

Fig. S4. Genomic loci of *C. elegans* and *C. briggsae fbn-1*

(A, B) Images taken from WormBase of the *C. elegans* (A) and *C. briggsae* (B) *fbn-1* genomic loci. RNAseq data are displayed below the pink gene diagrams (black lines and bars). RNAseq scores correspond to read numbers. The *C. elegans fbn-1* locus is enlarged to highlight the relevant region (red bracket), with introns 14–16 indicated. For *C. briggsae fbn-1*, the number of intronic in-frame stop codons is indicated (arrows). Note the absence of detected in-frame stop codons in intron 14 (blue arrow).(C) Available sequence on WormBase for intron 14 of *C. briggsae fbn-1*, which includes an unassigned 346-bp central region (gray background). The regions downstream of exon 14 (402 bp) and upstream of exon 15 (347 bp) form a continuous open reading frame (blue background). Yellow background indicates exons 14 and 15.

Biology Open • Supplementary information

File S1. Data for figure panels.

Click here to download File S1

File S2. FAM102A and WDR44 LC/MS unfiltered and filtered protein data.

Click here to download File S2

File S3. FAM102A and WDR44 LC/MS unfiltered peptide reads.

Click here to download File S3

File S4. Enhancer of Pin RNAi screen data.

Click here to download File S4

File S5. Strain list.

Click here to download File S5

Biology Open • Supplementary information

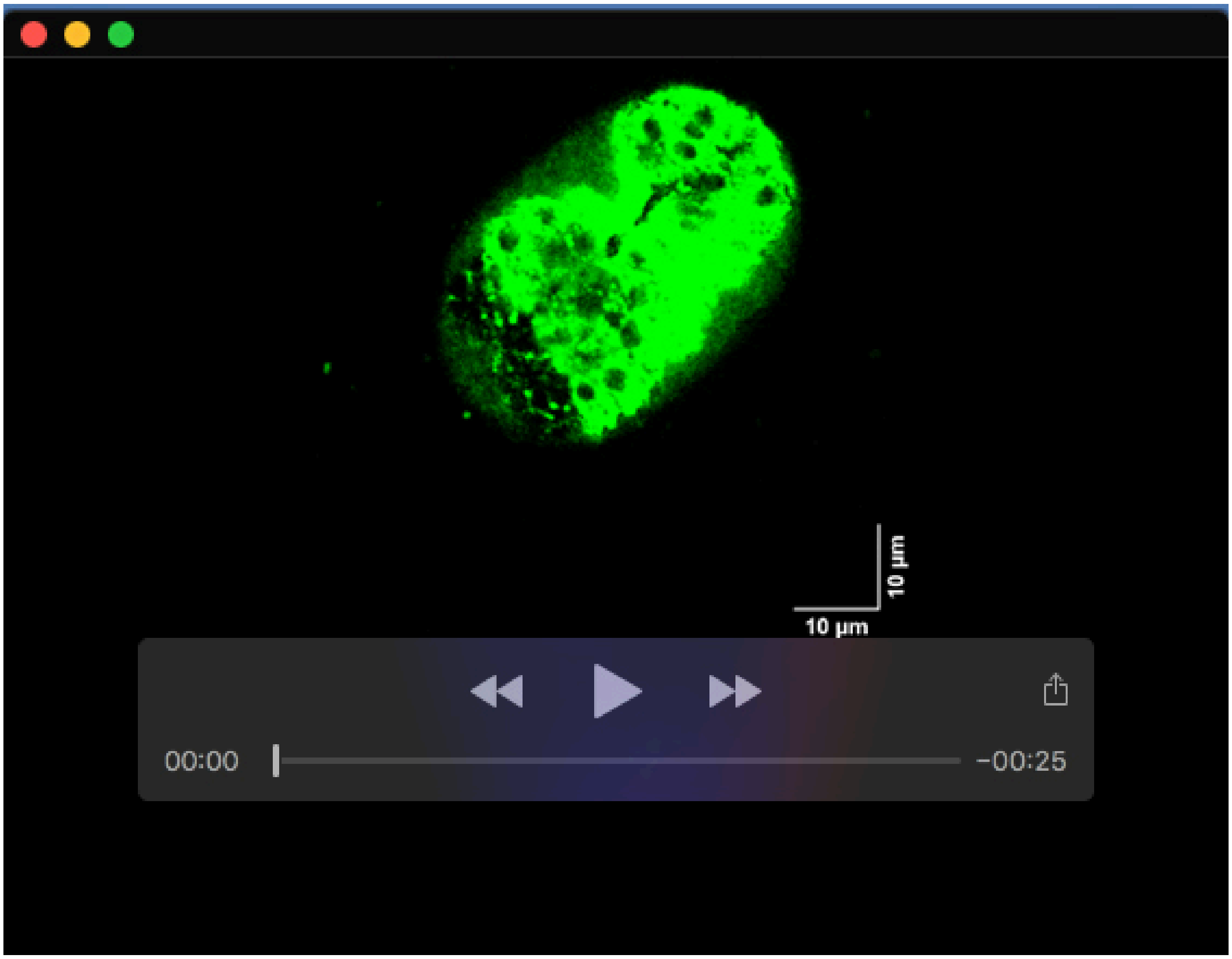

Movie 1. z-Stack movie of FBN-1::sfGFP in a bean-stage embryo.

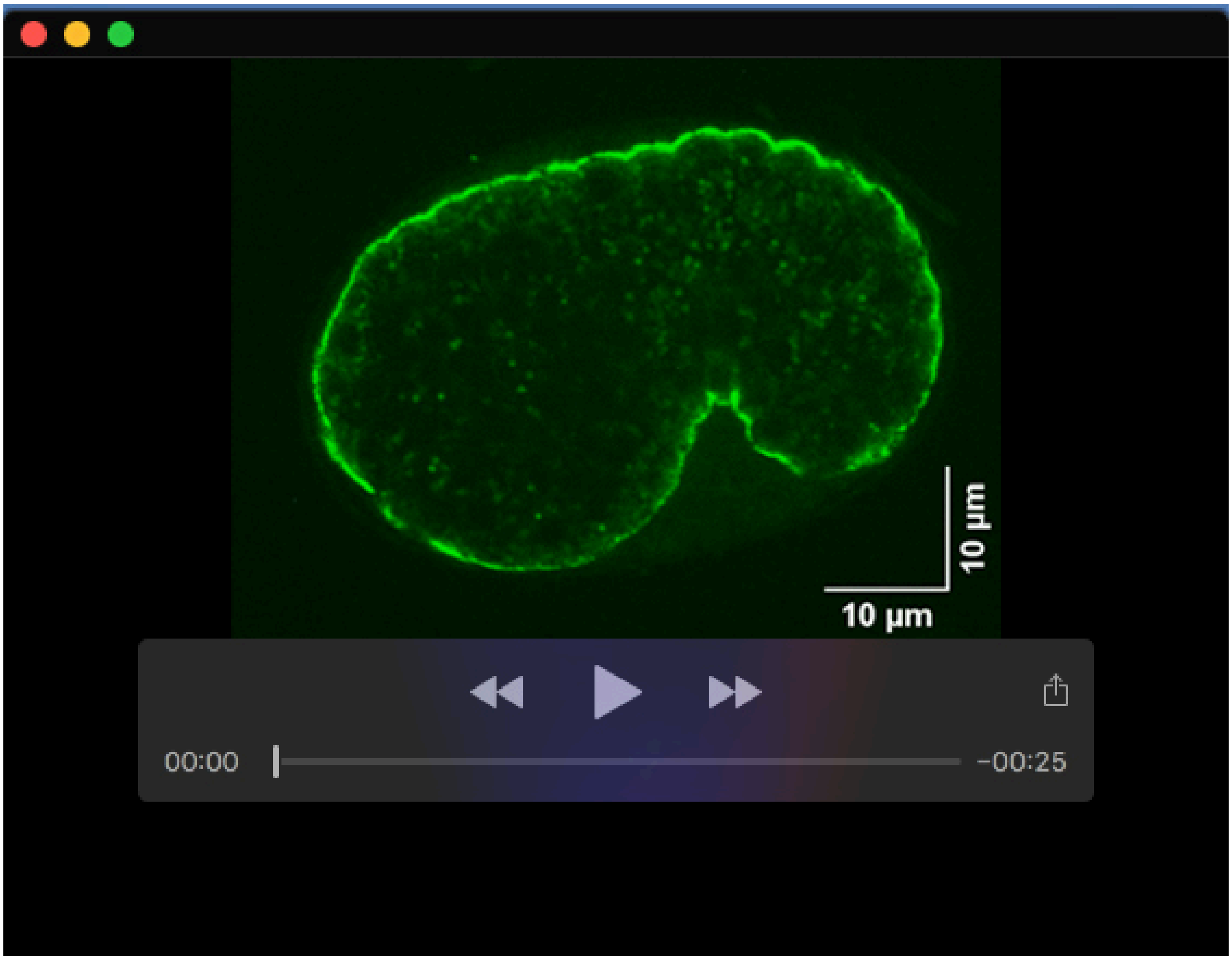

Movie 2. z-Stack movie of FBN-1::sfGFP in an early comma-stage embryo.

Biology Open • Supplementary information

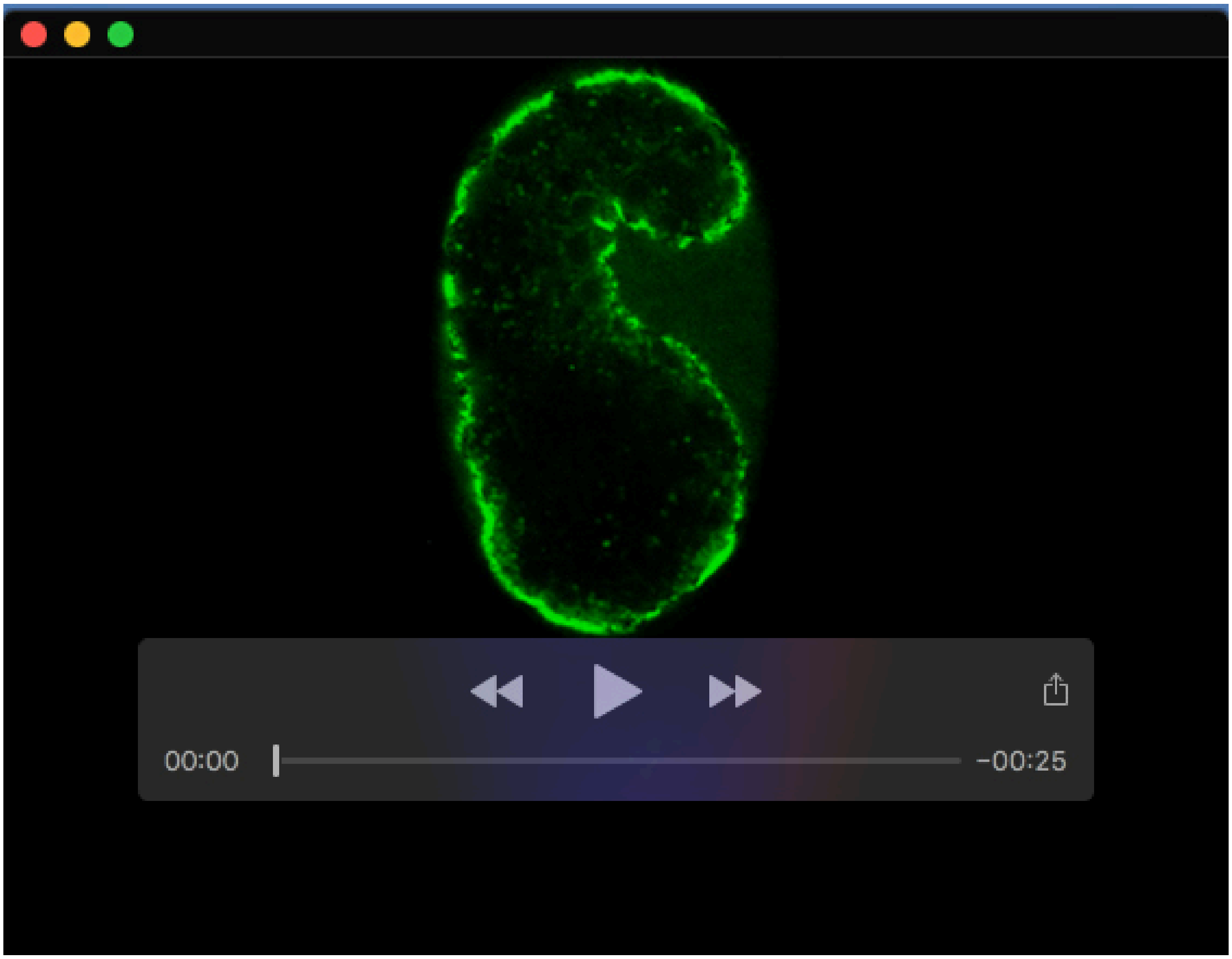

Movie 3. z-Stack movie of FBN-1::sfGFP in a late comma-stage embryo.
